# VALENF-Instrument-Based Nursing Assessment and Early Occurrence of Hospital-Acquired Pressure Injuries and Falls Among Hospitalized Adults

**DOI:** 10.3390/nursrep16030080

**Published:** 2026-02-25

**Authors:** David Luna-Aleixos, Víctor M. González-Chordá, Víctor Ortíz-Mallasén, Irene Llagostera-Reverter, Francisco H. Machancoses, Águeda Cervera-Gasch, Isabel Grao-Ros, María Isabel Orts-Cortés, María Jesús Valero-Chillerón

**Affiliations:** 1 Joint Research Unit NURSIA (“NURSing Care, Information Systems, Technology and Quality”) UJI-FISABIO, 46020 Valencia, Spain; luna_davale@gva.es (D.L.-A.); ortizv@uji.es (V.O.-M.); llagoste@uji.es (I.L.-R.); chillero@uji.es (M.J.V.-C.); 2 Hospital Universitario de La Plana, 12520 Vila-Real, Spain; isabel.grao@hospitalprovincial.es; 3 eNursys Research Group (Code 162), Foundation for the Promotion of Health and Biomedical Research in the Valencian Region (FISABIO), 46020 Valencia, Spain; 4 Nursing Research Group (GIENF Code 241), Nursing Department, Universitat Jaume I, 12071 Castellón de la Plana, Spain; 5 Nursing and Healthcare Research Unit (Investén-isciii), Instituto de Salud Carlos III, 28029 Madrid, Spain; isabel.orts@ua.es; 6 Network Biomedical Research Center on Frailty and Healthy Aging (CIBERFES), Instituto de Salud Carlos III, 28029 Madrid, Spain; 7 Predepartamental Unit of Medicine, Universitat Jaume I, 12071 Castellón de la Plana, Spain; herrerof@uji.es; 8 Department of Nursing, University of Alicante, 03690 Alicante, Spain; 9 Alicante Institute for Health and Biomedical Research (ISABIAL, Group 23), 03010 Alicante, Spain

**Keywords:** nursing, accidental falls, pressure ulcer, nursing assessment, hospitalization, survival analysis

## Abstract

**Background/Objectives:** Pressure injuries and falls are frequent hospital adverse events. Identifying high-risk periods may help guide preventive strategies. In this exploratory study, we aimed to estimate the time from hospital admission to the occurrence of pressure injuries and/or falls and analyze its relationship with the nursing assessment at admission. **Methods:** A longitudinal observational study was conducted with a systematic sample of 314 adult patients admitted between January and May 2024. Nursing assessment at admission was performed using the VALENF Instrument, which integrates functional capacity, pressure injury risk, and fall risk. Survival analysis was performed to describe the temporal distribution of adverse events and compare their occurrence across nursing assessment variables using the log-rank test. Poisson Generalized Linear Models were applied to explore associated factors. **Results:** Nineteen adverse events were recorded (15 pressure injuries and 4 falls). Twelve of the 19 total events (63%) occurred within the first five days of admission. Patients with lower functional capacity (log-rank *p* < 0.001) and high-pressure injury risk (log-rank *p* < 0.001) according to the VALENF Instrument, showed an earlier occurrence of new pressure injuries in the Kaplan–Meier analysis. Similarly, fall risk scores (log-rank *p* = 0.037) obtained with the same instrument were associated with falls. Patients classified as high risk for pressure injuries showed an approximately nine-fold higher incidence rate of developing new injuries (Wald χ^2^, *p* < 0.001), while urgent admission further increased this risk more than six-fold (Wald χ^2^, *p* = 0.015). **Conclusions:** In this exploratory study with a limited number of events, most adverse events occurred early during hospitalization. The findings suggest that early nursing assessment using the VALENF Instrument may help stratify patients for closer monitoring early in admission, pending confirmation in larger studies.

## 1. Introduction

Patient safety is a cornerstone of hospital care quality, given the range of risks that can compromise it [1]. Safety issues in inpatient units are multifactorial and encompass various adverse events, such as medication errors, healthcare-associated infections, pressure injuries, and falls [2], with the latter two being the most common in hospital settings [3]. These problems extend beyond the physical discomfort, harm, and potential long-term consequences they may cause to the affected patients.

On the one hand, pressure injuries are associated with an increased risk of healthcare-associated infections, prolonged hospital stays, pain, and disability [4]. These injuries have significant morbidity and mortality [5], accounting for approximately 60,000 deaths each year in the United States alone [6]. This represents a considerable additional cost for hospitals, which could be mitigated by implementing appropriate preventive care [7]. On the other hand, patient falls during hospitalization can have serious physical and psychological consequences. One in four falls results in injury, which is severe in approximately 10% of cases [8]. Beyond physical harm, a fall can lead to fear, distress, depression, and reduced physical activity in patients [9], thereby increasing healthcare costs and contributing to deteriorating functional capacity [10]. Moreover, low levels of functional capacity are associated with falls and with an increased risk of other adverse events, such as pressure injuries [11].

An appropriate nursing assessment enables the early identification of patients at higher risk of falls or pressure injuries and the implementation of preventive measures that promote patient safety [12]. To this end, validated assessment tools and clinical practice guidelines or protocols are usually available in healthcare institutions [13,14]. However, beyond identifying patients at risk, it is also essential to recognize the moment when these adverse events actually occur [15,16]. The literature consistently describes risk factors associated with these events, depending on a patient’s clinical condition [17], pharmacological treatment [18], and functional capacity [19,20]. Nevertheless, most available studies are based on retrospective analyses or cross-sectional designs, which limit the understanding of the dynamics and temporal component of falls or pressure injuries.

In this context, the VALENF Instrument (acronym derived from the Spanish term for Nursing Assessment) is an innovative tool designed to facilitate routine nursing assessment. Its development and validation, including psychometric properties and operational framework, have been described elsewhere [21,22,23]. This meta-instrument, composed of seven items, was derived through regression analyses aimed at estimating the scores of the Barthel Index, the Braden Scale, and the Downton Scale in a simplified and integrated format, using a parsimonious subset of items identified through stepwise modelling [21]. Importantly, this approach sought to reproduce the informational content of established instruments rather than to predict clinical events directly. Previous studies therefore focused on instrument-level agreement and classification performance relative to those tools [21,22]. The present study extends this line of work by exploring whether baseline VALENF-based assessments are associated with the subsequent in-hospital occurrence of pressure injuries and falls using time-to-event analyses. This does not imply diagnostic or predictive validation against clinical outcomes, but rather examines exploratory associations within routine clinical care.

Despite the availability of validated instruments for assessing functional capacity, fall risk, and pressure injury risk, current clinical practice often relies on multiple separate tools [24,25,26], increasing workload and reducing assessment efficiency [27]. Moreover, although prior research has identified risk factors for these adverse events, existing reviews have also reported information on time-to-event outcomes and temporal patterns during hospitalization [28,29]. *However, these syntheses describe overall incidence or static risk factors,* and evidence regarding the distribution of events across specific admission days remains heterogeneous across clinical settings. In particular, the potential clustering of pressure injuries and falls during the first days of hospitalization, and its relationship with baseline nursing assessment finding, has not been consistently examined in acute care cohorts.

A clearer description of early temporal distribution may help to inform prioritization of monitoring intensity, resource allocation, and preventive vigilance during the initial phase of admission, rather than assuming uniform risk throughout hospital stay. Therefore, the objective of this study was to estimate the time from hospital admission to the occurrence of pressure injuries and/or falls among adult patients admitted to medical-surgical hospitalization units and to analyze its relationship with the nursing assessment at admission.

## 2. Materials and Methods

### 2.1. Design and Setting

An observational, longitudinal, and prospective study was conducted. The STROBE guidelines for reporting observational studies were followed [30]. This study was carried out at Hospital Universitario de La Plana in Vila-real (Spain), the reference center for the Health Department of La Plana. The hospital has 258 beds and provides coverage for an approximate population of 190,000 inhabitants. Each year, the institution records around 11,000 admissions, 9000 surgical interventions, 65,000 emergency visits, and 200,000 outpatient consultations.

### 2.2. Participants and Sample

The study population consisted of patients admitted to the adult medical–surgical hospitalization units (Traumatology, Surgery/Gynecology, Cardiology/Gastroenterology, Neurology/Pulmonology, General Surgery, Internal Medicine). Special services (such as intensive care, emergency, operating room, and post-anesthesia care), home hospitalization, and maternal–child and obstetric–gynecological hospitalization units were excluded.

All patients aged 18 years or older, with an expected hospital stay of more than 48 h and a nursing assessment completed within the first 24 h after admission, were included in this study. Inclusion required the patient’s explicit consent, formalized by signing an informed consent form. The exclusion criteria were (i) patients admitted for care related to palliative processes and (ii) patients transferred from other units or hospitals, as it was not possible to obtain their assessments within the first 24 h after admission. It is necessary to clarify that patients admitted for palliative care were excluded because they represent a clinically distinct population with specific care goals, and their inclusion could have biased the analysis due to their higher baseline risk of pressure injuries.

Regarding the sample size, this study was derived from a secondary objective specified in the protocol of a larger project that was primarily designed to determine the diagnostic accuracy of the VALENF Instrument (trial registration number: SRCTN 17699562, 25 July 2023) [23]. Therefore, the same cohort of patients as in the diagnostic test study was used. The sample size was estimated at 280 participants using the Epidat program (version 4.0), based on a comparison of paired proportions. Specifically, pressure injuries were considered the main outcome, and the prevalence of 8.7%, as reported in Spanish hospitals by Pancorbo-Hidalgo et al. [31], was used as a reference. An expected sensitivity and specificity of 90% for the VALENF Instrument, a 95% confidence level, 80% power, and a 10% replacement rate were assumed, resulting in an estimated sample size of 280 participants. Recruitment was carried out using systematic sampling, including all patients admitted every five calendar days to the participating hospitalization units. This sample size calculation was performed for the primary diagnostic accuracy objective of the VALENF project. The present manuscript addresses a secondary, prospectively planned objective based on the same cohort. Given that the observed incidence of new pressure injuries (4.8%) and falls (1.3%) was lower than anticipated, the effective statistical power for this secondary time-to-event analysis is reduced and all multivariable models should be interpreted as exploratory.

### 2.3. Variables

The nursing assessment results obtained using the VALENF Instrument were collected [21,22]. This meta-tool, including seven items, integrates the assessment of functional capacity, the risk of pressure injuries, and the risk of falls and has demonstrated adequate structural validity, internal consistency, and inter-observer reliability [22]. In addition, age and sex were collected as sociodemographic variables due to their consistently reported association with both pressure injury and fall risk in hospitalized adults. Moreover, variables related to the care process, such as type of process (medical or surgical), type of admission (emergency or scheduled), hospitalization unit (Traumatology, Surgery/Gynecology, Cardiology/Gastroenterology, Neurology/Pulmonology, General Surgery, Internal Medicine), and main diagnoses according to the International Classification of Diseases, 10th Revision (ICD-10-CM, 2024) [32]. Based on these diagnoses, the Charlson Comorbidity Index was calculated [33,34].

Regarding the events of interest, the presence of a pressure injury at admission (yes/no) was recorded, as well as whether the admission was due to a fall (yes/no). Similarly, information was collected on the implementation of preventive measures for falls and pressure injuries (yes/no/unconfirmed). Only new hospital-acquired pressure injuries and in-hospital falls occurring after admission were considered incident events. Preventive measures were recorded descriptively as part of routine clinical care and should not be interpreted as exposures or indicators of intervention effectiveness, as their implementation is risk-driven and occurs after the initial clinical assessment. These measures were implemented by clinical nurses independently of the study and without knowledge of the assessments performed by the research team. For the outcome variables, if a pressure injury or a fall was identified during follow-up, the exact date of the event was retrieved from the electronic health record and recorded on the follow-up forms, allowing us to calculate the number of days from hospital admission to the occurrence of the first clinical event.

### 2.4. Data Collection

Data collection was carried out between January and May 2024, with patients followed longitudinally from admission. Members of the research team and clinical nurses from the participating hospital units were involved in the study. Before data collection, training sessions were conducted with the professionals involved to ensure the consistency and standardization of the information collected. These sessions focused on the data collection form and the software used to enter information into the electronic data capture system (Research Electronic Data Capture—REDCap) [35]. In addition, a 15-day pilot period was established to validate the data collection procedure and identify potential issues.

Participants were recruited periodically, every five calendar days, including only patients admitted within the previous 24 h. Subsequently, the patients were reassessed every five days until hospital discharge, at which point the final data collection was performed. The rotating five-day recruitment schedule reduced the likelihood of systematically biasing the sample according to recurring temporal admission patterns.

Sociodemographic and care-related variables were recorded only at baseline, whereas nursing assessments using the VALENF Instrument, along with variables related to the occurrence of pressure injuries and falls and the implementation of preventive measures, were collected at baseline, during periodic reassessments, and at the final evaluation upon discharge.

### 2.5. Data Analysis Procedures

A descriptive analysis of the study sample was performed according to the nature of each variable. Categorical variables were summarized using absolute and relative frequencies, whereas continuous variables were described using the mean and standard deviation. Following this initial analysis, the cumulative incidence and incidence rate of pressure injuries and falls were estimated globally and by unit, according to sociodemographic variables, care-related variables, and functional capacity. To categorize age and functional capacity, a cluster analysis was conducted to group the sample into homogeneous subgroups. First, an exploratory analysis was performed using the hierarchical (Ward’s) method to identify the most appropriate number of clusters, followed by the k-means method to define the boundaries of each category, without interpreting these as clinically established cut-off points. For the incidence rate, the duration (in days) of each care process was considered as the time at risk for developing any of the adverse events under study.

Secondly, a survival analysis was performed using the nonparametric Kaplan–Meier method. This analysis was used to explore the temporal evolution of pressure injuries and falls throughout the hospitalization period. Subsequently, survival curves for each event were compared according to the categorical variables included in this study using the log-rank test. Finally, to assess the association between functional capacity, the risk of pressure injury, and the risk of falls with the incidence of adverse events during hospitalization, Generalized Linear Models (GLMs) with a Poisson distribution and a logarithmic link function were fitted. Length of stay was included as an offset term to model incidence rates (IRR) based on time at risk. The outcomes were defined as the new hospital-acquired pressure injuries and in-hospital falls occurring after admission. Given the limited number of events (15 pressure injuries and 4 falls), all models were considered exploratory in nature, that is, aimed at exploring associations between baseline variables and event occurrence rather than at individual-level prediction. Accordingly, model complexity was intentionally restricted to avoid overparameterization given the number of observed events.

Potential covariates were selected using bivariate analyses (*p* < 0.20) and theoretical relevance [36]. Covariates that met these criteria were subsequently evaluated using the change-in-estimate criterion, considering any variable whose inclusion modified the coefficient of the main predictor by ≥10% as a confounding variable (on the logarithmic scale) [36]. These cut-off points are commonly used in exploratory multivariate analyses, as they help reduce residual confounding and improve model interpretability [37]. This selection strategy was applied within an exploratory framework and was not intended to derive a definitive or optimal model specification.

For each model, a Type III test of effects was calculated to assess the partial contribution of each covariate after adjusting for the others, using the Likelihood Ratio Chi-square test. In addition, B coefficients, incidence rate ratios (IRRs), and their corresponding 95% confidence intervals were estimated, disaggregated by the category of each variable, taking the lowest-risk category as the reference. This approach allowed for identifying the magnitude and direction of the effect of each covariate level on the incidence rate of the events, within an exploratory framework adjusted for exposure time. The scale parameter was estimated from the data using the deviance-based method to account for potential overdispersion in the count of adverse events. This approach provides more robust standard errors without affecting the direction or magnitude of incidence rate ratios.

Statistical analyses were performed using SPSS software, version 29.0.1.0 (IBM, Armonk, NY, USA), with a significance level set at *p* < 0.05.

### 2.6. Ethical Considerations

This study was designed in accordance with Organic Law 3/2018 of December 5, on Personal Data Protection and Guarantee of Digital Rights (Government of Spain, 2018), and with Regulation (EU) 2016/679 of the European Parliament and of the Council of 27 April 2016, on the protection of natural persons (European Parliament and Council of the European Union, 2016).

This study was authorized by the hospital management and approved by the Research Ethics Committee of Hospital Universitario de La Plana (code VALENF. 20 June 2023). All participants received an information sheet explaining the aim of the study, the procedures involved, and the potential implications for the healthcare system, along with information regarding their rights to access, rectification, and withdrawal without any impact on their clinical care. After receiving and reviewing this information, participants were given an informed consent form to sign prior to their inclusion in the study. The study did not involve any intervention beyond routine clinical practice and did not entail foreseeable risks or harm to participants. All data were anonymized prior to analysis, and confidentiality was strictly maintained throughout the study. Participation was voluntary and did not influence clinical care in any way.

## 3. Results

### 3.1. Descriptive Analysis of the Sample

A total of 365 participants were initially recruited. However, six of them (1.7%) were excluded because they lacked the cognitive capacity to sign the informed consent form and did not have a legal representative who could do so on their behalf. In addition, two participants (0.5%) were excluded because they were in a terminal condition. Moreover, 41 participants (11.2%) were excluded because they remained hospitalized for less than 48 h, and another 2 (0.5%) were excluded because they voluntarily decided to withdraw from the study. Consequently, the final sample consisted of 314 participants (86%). The mean length of stay was 7 days (SD = 5.6; median, 6 days). The minimum stay was 3 days, and the maximum was 39 days.

Table 1 presents a descriptive analysis of the sample according to sociodemographic and clinical variables, as well as their relationship with the occurrence of pressure injuries and/or falls during hospitalization. The sample consisted of 51% men (n = 160). A total of 49.7% (n = 156) of the participants were aged between 75 and 98 years. Medical processes predominated (83.4%; n = 262), as did emergency admissions (93.6%; n = 294). The hospitalization units that contributed the most participants to the sample were the Traumatology unit (24.2%; n = 76) and the Neurology/Pulmonology unit (23.6%; n = 74). Nearly half of the participants (47.8%; n = 150) were independent or mildly dependent at admission, whereas 69.7% (n = 219) presented a high level of comorbidity according to the Charlson Comorbidity Index.

Regarding pressure injuries, three patients were admitted with an active lesion, one of whom developed a new injury during hospitalization. In total, 15 pressure injuries (4.8%) were recorded throughout the hospital stay. The injuries occurred mainly in women (n = 10) and in the age group between 75 and 98 years (n = 10). Incidence was also higher among patients who underwent medical processes (n = 11) and those admitted as emergencies (n = 13). By unit, the highest numbers were observed in Neurology/Pulmonology (n = 5) and Traumatology (n = 4). Preventive measures to avoid pressure injuries were implemented in 32.5% (n = 102) of the patients upon hospital admission; among these patients, nine developed a pressure injury during their stay. Regarding incidence density, expressed as the number of events per 1000 person-days, the highest rate was observed among patients with severe dependence at admission (19 cases per 1000 person-days).

Regarding falls, four events (1.3%) were recorded during hospitalization, all in patients admitted as emergencies. Most falls took place among men (1.9%; n = 3). The incidence of falls was higher in medical processes (1.1%; n = 3). By unit, falls occurred mainly in the Surgery/Gynecology unit (5.8%; n = 3). Regarding preventive measures, 79% (n = 248) of the patients received interventions aimed at preventing falls. The incidence density of falls was concentrated in surgical units, particularly in the Surgery/Gynecology unit (8 cases per 1000 person-days).

### 3.2. Bivariate Analysis of Time-to-Event Outcomes (Pressure Injuries and Falls)

**Figure 1:**
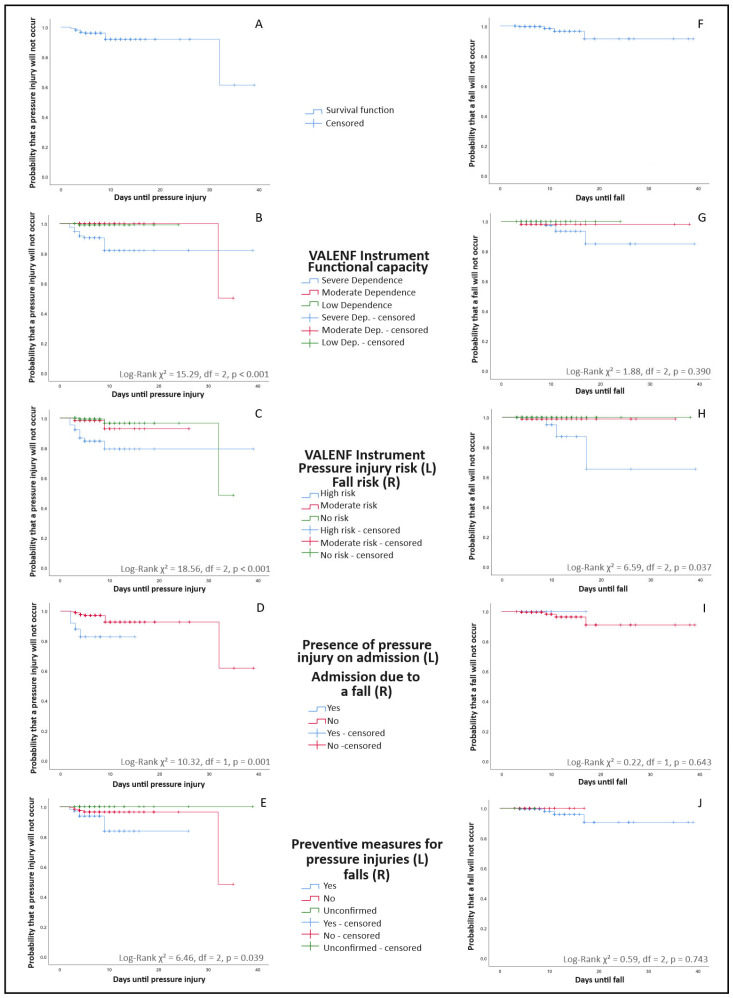
Kaplan–Meier survival curves for time-to-event outcomes (pressure injuries and falls) according to the results of the nursing assessment. Curves stratified by preventive measures reflect risk-driven care allocation (confounding by indication) and should not be interpreted as evidence of preventive intervention effectiveness. (**A**) Overall survival free of pressure injuries. (**B**) Survival free of pressure injuries stratified by functional capacity at admission. (**C**) Survival free of pressure injuries stratified by pressure injury risk level according to the VALENF Instrument. (**D**) Survival free of pressure injuries stratified by the presence of pressure injury at admission. (**E**) Survival free of pressure injuries stratified by implementation of preventive measures at admission. (**F**) Overall survival free of falls. (**G**) Survival free of falls stratified by functional capacity at admission. (**H**) Survival free of falls stratified by fall risk level according to the VALENF Instrument. (**I**) Survival free of falls stratified by admission due to a previous fall. (**J**) Survival free of falls stratified by implementation of fall-preventive measures at admission.

Figure 1 shows the survival curves corresponding to the occurrence of pressure injuries (graphs A–E) and falls (graphs F–J) from hospital admission, according to the patients’ initial assessments. The ordinate axis represents the cumulative probability of the event (pressure injury or fall) not occurring, whereas the abscissa axis represents the number of days from hospital admission until the event occurred or until discharge. Each drop in the curve reflects the occurrence of an event (pressure injury or fall) in proportion to the number of patients at risk at that time. As shown, pressure injuries occurred on days 2, 2, 2, 3, 3, 3, 4, 4, 4, 4, 5, 9, 9, 9, and 32 (Figure 1A), while falls occurred on days 4, 9, 11, and 17 after admission (Figure 1F). Therefore, 63.16% of all the adverse events (n = 12) occurred within the first five days of hospitalization. Patients discharged without a pressure injury (95.2%, n = 299) or a fall (98.7%, n = 310) are represented as censored cases, marked by perpendicular lines along the curve trajectory. Additional analyses for age, sex, type of admission, type of process, hospitalization unit, and comorbidity (Charlson Comorbidity Index) showed no statistically significant associations (*p* > 0.05) with the development of either pressure injuries or falls.

Table 2 presents the descriptive data corresponding to the curves shown in Figure 1. Specifically, regarding pressure injuries, a significant association was observed between functional capacity at admission and the occurrence of pressure injuries (*p* < 0.001). In particular, 86.7% (n = 13) of the identified pressure injuries occurred in patients with severe dependence, with a median onset around the fourth day of hospitalization (Figure 1B). Among the pressure injuries that developed during hospitalization, the VALENF Instrument identified 66.7% (n = 10) of the patients as being at high risk of developing a pressure injury, whereas 20% (n = 3) were classified as not at risk (Figure 1C, *p* < 0.001). Moreover, 73% (n = 11) of the injuries occurred in patients who had no pressure injuries at admission, whereas among those who already had one at admission, 16% (n = 4) developed a new lesion during hospitalization (Figure 1D, *p* = 0.001). Regarding preventive measures, 40% (n = 6) of the pressure injuries occurred in patients for whom no preventive interventions were implemented from admission, and these appeared during the first week of hospitalization. Among patients who received preventive measures from the first assessment, 8.8% (n = 9) developed a pressure injury (Figure 1E, *p* = 0.039).

Similarly, as shown in Figure 1B, even patients with moderate dependence developed pressure injuries during prolonged hospitalizations, particularly after the 30th day of admission. Likewise, Figure 1C shows that some patients developed pressure injuries despite not presenting an apparent risk at admission, underscoring the importance of continuous reassessment.

Only four fall events were identified during follow-up (Figure 1F). Of these, three occurred in patients with severe dependence at admission (Figure 1G, *p* = 0.390) and in those classified as being at high risk of falls according to the VALENF Instrument assessment (Figure 1H, *p* = 0.037). In both cases, the median time to fall was eleven days. None of the events occurred in patients admitted due to a previous fall (Figure 1I, *p* = 0.643), and all falls (n = 4) occurred in patients for whom fall-prevention measures were implemented from hospital admission (Figure 1J, *p* = 0.743).

### 3.3. Multivariate Analysis of Event Incidence Rates (Pressure Injuries and Falls)

Poisson Generalized Linear Models (GLM) were fitted to estimate the incidence rate of new pressure injuries and in-hospital falls. The following tables (Table 3 and Table 4) present the results of these models, including the Type III Likelihood Ratio Chi-square tests, which were used to assess the overall contribution of each predictor or covariate to the model, and the parameter estimates (B coefficients), which quantify the effect of each category relative to the reference group. The incidence rate ratio (IRR = Exp(B)) expresses the multiplicative change in the expected event rate associated with each category, where values greater than 1 indicate an increased incidence rate and values below 1 indicate a decreased incidence rate. Confidence intervals (95% CIs) were computed for each IRR to assess the precision and statistical significance of the estimates. The intercept represents the estimated baseline incidence rate for the reference categories of all predictors, serving as the model’s reference level for comparison.

For pressure injuries (Table 3), four generalized linear Poisson models were fitted. Two models used functional capacity as the main predictor (a base model and a model adjusted for pressure ulcer on admission), and two models used pressure injury risk as the main predictor (a base model and a model adjusted for type of admission). To ensure model convergence and interpretability, both predictors were dichotomized. In both cases, estimations were obtained by including one main predictor adjusted for one covariate, rather than using the base models.

As a preliminary step, a base Poisson model including only the main predictor was fitted without covariates. In this base model, patients with severe functional dependence showed a markedly higher incidence rate of new pressure injuries compared with those with no or mild dependence (IRR = 10.38; 95% CI: 2.40–44.98; *p* = 0.002). When pressure injury on admission was added as a covariate, the association between severe functional dependence and incident pressure injury remained statistically significant, although the effect size was attenuated by 13.48% relative to the base model, suggesting that this covariate may act as a potential confounder. The adjusted specification yielded a lower AIC and a higher omnibus χ^2^, showing a comparatively improved fit within this exploratory framework. Accordingly, the adjusted model was retained for exploratory interpretation. Considering these results, the adjusted model was retained as the preferred specification. Thus, patients with severe functional dependence had almost a ninefold higher incidence of developing a new pressure injury during hospitalization (Table 3).

Table 3 also presents a second pair of models in which high pressure injury risk (yes/no) was used as the main predictor. A base Poisson model including only this predictor was first fitted without covariates. In this unadjusted model, patients classified as being at high risk had a 7.44 times higher incidence rate of new pressure injuries than those at moderate or lower risk (IRR = 7.44; 95% CI: 2.54–21.81; *p* = 0.001). When the type of admission (urgent vs. scheduled) was introduced as a covariate, the association remained significant and the IRR for high risk increased to 9.83 (95% CI: 2.95–32.80; *p* < 0.001), representing a 32.1% change relative to the base model. In the adjusted model, urgent admission was itself significantly associated with pressure injury incidence (IRR = 6.37; 95% CI: 1.44–28.25; *p* = 0.015). Including this covariate improved the overall model fit (AIC decreased from 123.47 to 121.85; omnibus χ^2^ increased from 10.76 to 13.46), and the adjusted specification was therefore retained for exploratory interpretation (Table 3). Thus, patients classified as being at high risk of pressure injury had almost a tenfold higher incidence of developing a new lesion during hospitalization, and urgent admission was independently associated with a sixfold higher incidence.

Finally, Table 4 presents the Generalized Linear Model (GLM) with a Poisson distribution and a log link function fitted to estimate the association between fall risk at admission and the incidence of in-hospital falls. The predictor was entered as a three-level categorical factor (low [reference], moderate, and high risk). Given the very small number of events (n = 4), no covariates were included. The model showed an acceptable overall fit (Omnibus χ^2^(2) = 8.699; *p* = 0.013). Compared with patients classified as low-risk, those categorized as moderate- and high-fall-risk presented lower observed incidence rates of falls (IRR = 0.817; 95% CI: 0.678–0.983; *p* = 0.032 and IRR = 0.808; 95% CI: 0.650–1.004; *p* = 0.055, respectively). All recorded falls occurred in patients who already had fall-prevention interventions implemented at admission. Therefore, the model describes the observed fall incidence within the study context and should not be interpreted as estimating baseline fall risk.

## 4. Discussion

The results of this study provide a descriptive overview of the temporal distribution of pressure injuries and falls during hospitalization, and their association with baseline nursing assessment variables. This knowledge may inform future research on the timing and intensity of preventive interventions. In this regard, the initial nursing assessment using the VALENF Instrument showed the most consistent association with the occurrence of pressure injuries and falls during hospitalization. Functional capacity, risk of pressure injuries, and risk of falls, as assessed through the VALENF Instrument, showed a significant relationship with the incidence of adverse events, within this sample, which should not be interpreted as evidence of clinical effectiveness. However, these associations should be interpreted as exploratory, since this secondary analysis was not designed to determine predictive performance or diagnostic accuracy, and therefore the present findings do not establish clinical effectiveness or predictive validity of the instrument’s capacity to prevent or detect adverse events [23].

It was also observed that most adverse events were concentrated within the first five days after hospital admission, consistent with previous research [19]. This pattern does not seem to be explained solely by the length of stay, which averages around 6.5 days in acute care hospitals in Spain (maximum: 10.53; minimum: 4.33) [38], but rather may reflect an initial period of increased observed clinical vulnerability. This finding highlights the potential relevance of appropriate nursing assessments at the beginning of hospitalization to distinguish between different patient risk profiles [17,19], thereby allowing early characterization of risk within routine care, without establishing causal effects or preventive effectiveness [39,40].

In this context, the close relationship observed between clinical deterioration and the occurrence of adverse events is consistent with the literature on hospitalization-associated disability (HAD), which indicates that functional decline may begin at very early stages of the hospital stay, even within the first 24 h [41,42], which may underscore the potential relevance of early preventive attention from the onset of care rather than establish direct intervention effects. Although this observation may raise questions regarding the appropriateness and effectiveness of the safety measures implemented [43,44], it should be interpreted with caution. Preventive measures are typically initiated in response to perceived or assessed risk and therefore cannot be disentangled from baseline vulnerability in the present study design (confounding by indication). These findings reflect risk-driven care allocation rather than failure of preventive strategies. Accordingly, our data do not allow conclusions regarding the effectiveness of the preventive interventions implemented in routine practice, underscoring the need for adequately powered pragmatic studies and trials to evaluate preventive effectiveness [8,45].

Among the factors associated with the occurrence of pressure injuries, functional capacity emerged as one of the variables most consistently associated with the outcome: the lower the functional capacity, the higher the likelihood of developing pressure injuries, in agreement with previous research [19]. This finding aligns with the fact that reduced mobility increases the duration of sustained pressure on vulnerable body areas, limits the ability to respond to physical discomfort, and decreases the effectiveness of certain preventive interventions [46,47]. Moreover, functional dependence reflects a general state of greater clinical deterioration and frailty, which may be associated with a profile of high vulnerability to pressure injuries [48].

The presence of pre-existing pressure injuries at admission was also significantly associated with the development of new lesions during hospitalization, which may indicate that pre-existing injuries may serve as a marker of skin fragility and persistent vulnerability [49,50]. These findings are consistent with recommendations for closer monitoring in patients with existing lesions [50]. However, when this variable was included in the multivariate model, the association lost statistical significance, probably due to the small number of patients in this condition within the sample. It would therefore reasonable to examine this variable in future studies with a larger sample size, given its potential relevance.

It is also worth noting that the literature highlights not only the level of deterioration at a given point in time but also the rate at which it occurs. Some studies suggest that a rapid decline in functional status increases clinical vulnerability more markedly than a low but stable functional level over time [51,52]. The potential influence of this dynamic was reflected in two situations observed in the present study. First, the combination of emergency admission and a high risk of pressure injuries was associated with a markedly higher incidence rate of pressure injuries, in line with research indicating that acute processes can disrupt self-care, exacerbate frailty, and increase care demands during the first days of hospitalization [53,54,55]. This finding may highlight the potential relevance of implementing preventive measures from the time of admission, considering that the functional decline observed upon hospital arrival likely began before hospitalization [56,57]. Second, one patient developed a pressure injury after approximately one month of hospitalization, despite being initially assessed as not at risk, with moderate dependence, no previous lesions, and no preventive measures implemented. This observation illustrates that, in this sample, some patients assessed as low or no risk, and with prolonged hospital stays, may remain vulnerable to unanticipated deterioration, rather than indicating systematic gaps in preventive care [50]. Consequently, this suggests the potential value of periodic reassessments of each patient’s condition to promptly identify clinical changes that may increase the risk of pressure injuries [58]. Nevertheless, establishing evidence-based reassessment intervals remains a knowledge gap in nursing practice.

Regarding falls, no significant differences in incidence density were observed between older patients and those with lower functional capacity. This result may be consistent with the possibility that the most fragile or high-risk patients bedridden, which, while reducing the likelihood of falls [59], also limits mobility and may have adverse effects on their physical and emotional well-being [60]. However, the use of mobility restrictions and other specific preventive practices were not directly measured in this study and should be examined in future research to better understand their influence on fall incidence.

Conversely, in patients with a moderate degree of dependence or in middle-aged groups (52–74 years), the combination of a certain level of physical autonomy with an inadequate perception of risk may be associated with a higher incidence of falls. In this context, several studies have reported that older adults tend to overestimate their mobility capacity [61,62], particularly men [63]. This may be consistent with higher incidence of falls observed among male patients [64], in line with the results of the present study. Therefore, future research may examine whether incorporating risk perception into preventive strategies is associated with differences in observed fall incidence [65].

In addition to all the aforementioned consequences, the fact that most falls in our study occurred after the first five days of hospitalization may indicate that preventive vigilance should not be limited to the early phase of admission. To achieve this, accurate identification of patients at higher risk may be relevant within routine care, although these findings should be interpreted within the exploratory framework of the present study.

In relation to this aspect, the only study variable that showed a statistically significant association with the occurrence of falls was the fall risk assessment measured using the VALENF Instrument. It is important to note that no patients classified as having no risk or moderate risk experienced falls during their hospitalization. This pattern may be consistent with the potential role of the instrument in characterizing risk profiles during routine nursing assessment, as highlighted in previous literature [66]. However, these findings cannot be interpreted as evidence of predictive accuracy, as this study was neither designed nor powered to evaluate diagnostic performance. Accordingly, the results should be understood as preliminary indicators that warrant further investigation in dedicated diagnostic accuracy studies and in studies evaluating how risk assessment tools translate into effective preventive care.

### Limitations

This study has several limitations. First, the sample size was not determined for this specific objective, and the number of adverse events observed was low, which limits the precision of the estimates and prevents more complex multivariate analyses, thereby constraining the ability to explore the influence of other potentially relevant demographic or clinical variables. Moreover, given the limited number of observed events, the study had reduced capacity to detect confounding effects. The absence of statistically significant associations for certain clinical variables, including comorbidity burden, should therefore not be interpreted as evidence of no confounding.

Second, although the events were monitored prospectively and following a standardized procedure, their low incidence reduces the stability of the results. Furthermore, the incidence of new pressure injuries and falls was lower than anticipated in the original sample size calculation, resulting in a limited number of events (15 pressure injuries and 4 falls). This shortens the achieved statistical power and widens the confidence intervals, so all results from the multivariable models should be interpreted as exploratory and hypothesis-generating rather than confirmatory. Nevertheless, the findings are consistent with the variability reported in the literature regarding the frequency of falls and pressure injuries [67].

Third, this study was conducted in a single hospital, which limits the generalizability of the findings to other settings with different patient profiles, preventive protocols, or organizational structures. Finally, the VALENF risk categories used in this study were derived from the correspondence with the original assessment tools and were applied only at baseline. As a result, the development of pressure injuries or falls in some patients initially classified as low or no risk should not be interpreted as misclassification, but rather as a reflection of the dynamic clinical deterioration that may occur during hospitalization and the limitations of relying on a single initial assessment. Moreover, the predominance of emergency admissions and medical care processes reduces variability in structural characteristics and limits the interpretability of unit- or admission-type comparisons.

Additionally, selection bias cannot be excluded. Inclusion required informed consent and an expected hospital stay longer than 48 h, which may have resulted in the underrepresentation of patients with severe cognitive impairment or very short admissions. These factors could influence the observed incidence and timing of events. Although reassessments were conducted at predefined intervals, the exact date of event occurrence was recorded from clinical documentation; nevertheless, some degree of timing misclassification cannot be entirely ruled out. Finally, given the limited number of events and multiple exploratory comparisons performed, the possibility of model instability and type I error should be considered when interpreting the findings.

Finally, because preventive measures were implemented as part of routine clinical care in a risk-driven manner, associations involving preventive measures are subject to confounding by indication and were therefore interpreted descriptively. Furthermore, it should be considered that there was a real risk of contamination, meaning the nurses may have modified their routines due to the presence of the researchers.

Despite these limitations, the systematic follow-up of adverse events provides a solid foundation for the exploratory associations presented and underscores the need for studies with larger samples to confirm these preliminary results and advance the development of predictive models. Moreover, future studies incorporating scheduled reassessments and specifically designed diagnostic accuracy analyses are needed to determine the optimal thresholds and the predictive performance of the VALENF Instrument. Finally, pragmatic studies assessing how preventive interventions are implemented in real clinical contexts would help identify barriers and enabling factors for improving care quality.

## 5. Conclusions

The exploratory findings of this study indicate that early nursing assessments were associated with differences in observed risk profiles during hospitalization, although their direct impact on prevention requires confirmation in adequately powered prospective studies. The use of the VALENF Instrument within the first 24 h of admission classified patients into categories that showed differential incidence of pressure injuries and falls, and these assessments were consistently associated with the incidence of in-hospital events, within this sample. In this regard, the findings suggest a potential role for structured early nursing assessment as a stratification approach within an exploratory framework. Additionally, the first five days of hospitalization were observed to show a higher concentration of events in this cohort, although this temporal pattern should be interpreted cautiously given the limited number of events and potential influence of follow-up structure. In addition, they highlight the need for future studies with larger samples to evaluate the effectiveness of current preventive interventions and test whether VALENF-guided strategies improve patient outcomes.

## Figures and Tables

**Table 1:** Sociodemographic and clinical characteristics of the sample and their relationship with pressure injuries and/or falls observed during hospitalization (n = 314).

	Global	Pressure Injuries		Falls	
	%(n) ^1^	CI (n) ^2^	ID ^3^	CI (n) ^2^	ID ^3^
	100 (314)	4.8 (15)	7	1.3 (4)	2
Sex					
Male	51 (160)	3.1 (5)	5	1.9 (3)	3
Female	49 (154)	6.5 (10)	4	0.6 (1)	1
Age					
19–51 years	13.4 (42)	0 (0)	0	0 (0)	0
52–74 years	36.9 (116)	4.3 (5)	6	1.7 (2)	2
75–98 years	49.7 (156)	6.4 (10)	10	1.3 (2)	2
Process type					
Medical	83.4 (262)	4.2 (11)	6	1.1 (3)	2
Surgical	16.6 (52)	7.7 (4)	12	1.9 (1)	3
Admission type					
Scheduled	6.4 (20)	10 (2)	17	0 (0)	0
Emergency	93.6 (294)	4.4 (13)	6	1.3 (4)	2
Hospitalization unit					
Traumatology	24.2 (76)	5.3 (4)	8	0 (0)	0
Surgery/Gynecology	16.6 (52)	5.8 (3)	8	5.8 (3)	8
Cardio/Gastroenterology	9.9 (31)	0 (0)	0	0 (0)	0
Neuro/Pulmonology	23.6 (74)	6.8 (5)	9	0 (0)	0
General surgery	17.5 (55)	3.6 (2)	6	1.8 (1)	3
Internal medicine	8.3 (26)	3.8 (1)	5	0 (0)	0
Barthel index categories at admission					
Slight (80–100)	47.8 (150)	0.7 (1)	1	(0)	0
Moderate (35–75)	26.4 (83)	3.6 (3)	5	2.4 (2)	3
Severe (0–30)	25.8 (81)	13.6 (11)	19	2.5 (2)	3
Charlson Index					
Absence (0–1)	21.7 (68)	2.9 (2)	4	0 (0)	0
Low (2)	8.6 (27)	3.7 (1)	6	0 (0)	0
High (≥3)	69.7 (219)	5.5 (12)	8	1.8 (4)	2
Pressure injury on admission					
	1.0 (3)	33.3 (1)	91	0 (0)	0
Admission motivated by fall					
	0.3 (1)	0 (0)	0	0 (0)	0
Preventive measures for pressure injuries					
Applied	32.5 (102)	8.82 (9)	13		
Not Applied	54.5 (171)	3.51 (6)	5		
Unconfirmed	13.1 (41)	0 (0)	0		
Preventive measures for falls					
Applied	79 (248)			1.61 (4)	2
Not Applied	19.1 (60)			0 (0)	0
Unconfirmed	1.9 (6)			0 (0)	0

^1^ Percentage by column (sample); ^2^ percentage cumulative incidence (sample); ^3^ incidence density per 1000 person-days.

**Table 2:** Comparative survival analysis of time-to-event outcomes (pressure injuries and falls) according to the results of the nursing assessment using the Kaplan–Meier method and log-rank test.

Pressure Injuries	Events				Censored		
	n ^1^	% ^2^	Median ^3^	Range (IQR) ^4^	n ^1^	% ^2^	*p* ^5^
VALENF Instrument—Functional capacity							<0.001
Severe dependence	13	11.4	4	7 (5)	101	88.6	
Moderate dependence	1	1.6	32	- (-)	61	98.4	
Low dependence	1	0.7	4	- (-)	137	99.3	
VALENF Instrument—Pressure injury risk							<0.001
High risk	10	15.6	3.5	7 (2)	54	84.4	
Moderate risk	2	3.4	6	6 (-)	56	96.6	
No risk	3	1.6	9	28 (-)	189	98.4	
Presence of pressure injury on admission							0.001
Yes	4	16	2.5	2 (2)	21	84	
No	11	3.8	4	30 (6)	278	96.2	
Preventive measures for pressure injuries							0.039
Yes	9	8.8	4	7 (7)	93	91.2	
No	6	3.5	3.5	30 (9)	165	96.5	
Unconfirmed	-	-	-	-	41	100	
**Falls**	**Events**				**Censored**		
	**n ^1^**	**%^2^**	**median ^3^**	**range (IQR) ^4^**	**n ^1^**	**% ^2^**	***p* ^5^**
VALENF Instrument—Functional capacity							0.390
Severe dependence	3	2.6	11	8 (-)	111	97.4	
Moderate dependence	1	1.6	4	- (-)	61	98.4	
Low dependence	-	-	-	- (-)	138	100	
VALENF Instrument—Fall risk							0.037
High risk	3	4.6	11	8 (-)	62	95.4	
Moderate risk	1	1	4	- (-)	100	99	
No risk	-	-	-	- (-)	148	100	
Admission due to a fall							0.643
Yes	-	-	-	-	25	100	
No	4	1.4	10	13 (10)	285	98.6	
Preventive measures for falls							0.743
Yes	4	1.6	10	13(10)	244	98.4	
No	-	-	-	- (-)	60	100	
Unconfirmed	-	-	-	- (-)	6	100	

^1^ Absolute frequencies; ^2^ relative frequencies; ^3^ estimate of the day on which the events take place (median); ^4^ Min–Max Range (interquartile range); ^5^ log-rank (Mantel–Cox).

**Table 3:** Generalized Linear Models (Poisson regression) for pressure injury incidence according to functional capacity and pressure injury risk (VALENF Instrument).

		(LRT χ ^2^; df; *p*) ^1^	B ^2^	(Wald χ ^2^; df; *p*) ^3^	IRR (95% CI) ^4^
Predictor variable: functional capacity (VALENF Instrument)					
Functional Capacity (base model)	Intercept	(2777.94; 1; <0.001)	−6.49	(87.79; 1; <0.001)	0.002 (0.000–0.006)
	Severe dependence	(10.47; 2; 0.005)	2.34	(9.79; 1; 0.002)	10.38 (2.4–44.98)
	Moderate or low dependence		0		
	Scale parameter		1.319		
	Model summary	AIC = 122.77; Omnibus Chi-square = 11.36 (df = 1, *p* < 0.001).			
Functional Capacity adjusted for covariate: pressure injury on admission	Intercept	(3121.23; 1; 0.001)	−4.350	(11.55; 1; 0.001)	0.01 (0.001–0.159)
	Severe dependence	(9.59; 1; 0.002)	2.195	(8.63; 1; 0.003)	8.98 (2.08–38.86)
	Moderate or low dependence		0		1
	Pressure injury on admission	(2.27; 1; 0.132)	−1.108	(3.44; 1; 0.064)	0.33 (0.102–1.066)
	Scale parameter		1.286		
	Model summary	AIC = 121.85; Omnibus Chi-square = 13.92 (df = 2, *p* < 0.001).			
		**(LRT χ ^2^; df; *p*) ^1^**	**B ^2^**	**(Wald χ ^2^; df; *p*) ^3^**	**IRR (95% CI) ^4^**
Predictor variable: risk of pressure injury (VALENF Instrument)					
Pressure injury risk (base model)	Intercept	(2509.43; 1; <0.001)	−5.825	(176.28; 1; <0.001)	0.003 (0.001–0.007)
	High risk of pressure injury	(10.76; 1; <0.001)	2.007	(13.39; 1; <0.001)	7.44 (2.54–21.81)
	Moderate or risk of pressure injury		0		
	Scale parameter		1.33		
	Model summary	AIC = 123.466; Omnibus Chi-square = 10.76 (df = 1, *p* = 0.001)			
Pressure injury risk adjusted for covariate: Admission type	Intercept	(2490.4; 1; <0.001)	−7.989	(46.68; 1; <0.001)	0.000 (0.000034–0.003)
	High risk of pressure injury	(12.5; 1; <0.001)	2.285	(13.82; 1; <0.001)	9.83 (2.95–32.80)
	Moderate or risk of pressure injury		0		1
	Urgent admission	(2.69; 1; 0.101)	1.851	(5.93; 1; 0.015)	6.37 (1.44–28.25)
	Scale parameter		1.330		
	Model summary	AIC = 121.85; Omnibus Chi-square = 13.46 (df = 2, *p* = 0.001).			

^1^ (Likelihood Ratio Chi-square test; degrees of freedom; *p*-value); ^2^ B coefficient; ^3^ (Wald Chi-square test; degrees of freedom; *p*-value); ^4^ incidence rate ratio (95% confidence interval).

**Table 4:** Generalized Linear Models (Poisson regression) for fall incidence according to fall risk (VALENF Instrument).

Predictor Variable: Fall Risk (VALENF Instrument)			(LRT χ ^2^; df; *p*) ^1^	B ^2^	(Wald χ ^2^; df; *p*) ^3^	IRR (95% CI) ^4^
Intercept			(2161.95; 1; <0.001)	−1.185	(385.7; 1; <0.001)	0.31 (0.27–0.34)
High fall risk			(8.7; 2; 0.013)	−0.213	(3.69; 1; 0.055)	0.808 (0.65–1.00)
Moderate fall risk				−0.203	(4.58; 1; 0.032)	0.817 (0.68–0.98)
Low fall risk				0		1
Scale parameter				0.761		
	Model summary		Omnibus Chi-square = 8.699 (df = 2, *p* = 0.013).			

^1^ (Likelihood Ratio Chi-square test; degrees of freedom; *p*-value); ^2^ B coefficient; ^3^ (Wald Chi-square test; degrees of freedom; *p*-value); ^4^ incidence rate ratio (95% confidence interval).

## Data Availability

The database is available in an open repository. You can access the data through the following link: http://hdl.handle.net/10234/734860 (accessed on 9 June 2025).

## Public Involvement Statement

No public involvement in any aspect of this research.

## Guidelines and Standards Statement

This manuscript was drafted against the STROBE guidelines for observational research.
